# Complex receptor-ligand dynamics control the response of the VEGF system to protease injury

**DOI:** 10.1186/1752-0509-5-170

**Published:** 2011-10-21

**Authors:** Kimberly Forsten-Williams, Elma Kurtagic, Matthew A Nugent

**Affiliations:** 1 Department of Chemical Engineering, Virginia Polytechnic Institute & State University Blacksburg, VA 24061, USA; 2 Department of Biochemistry, Boston University School of Medicine, Boston, MA, USA; 3 Departments of Biochemistry & Ophthalmology, Boston University School of Medicine, Department of Biomedical Engineering, Boston University, Boston, MA, USA

## Abstract

**Background:**

Vascular homeostasis and response to injury are dependent on the coordinated activity of growth factors such as vascular endothelial growth factor-A (VEGF). VEGF signaling is mediated by VEGF receptors 1 (VEGFR1) and 2 (VEGFR2). VEGF also binds to extracellular matrix (ECM) and neuropilin (NP), a cell surface glycoprotein that enhances VEGF binding to VEGFR2 while inhibiting VEGF-VEGFR1 interactions. Proteases such as neutrophil elastase release VEGF bound to ECM; however, this results in proteolytic processing of VEGF to a smaller species termed VEGF fragment (VEGFf). We hypothesized that the generation and presence of VEGFf would have significant effects on the binding distribution of VEGF.

**Results:**

We show that VEGFf, unlike VEGF, does not bind ECM, fibronectin, or NP-1. Using computational simulations, we find that excess VEGFf can lead to increased binding of VEGF to VEGFR2 through VEGFf binding to VEGFR1 and subsequent liberation of NP-1. We show experimentally that VEGF-induced migration has a biphasic response to conversion of VEGF to VEGFf. Simulations suggest that a simple change in VEGFR1 or VEGFR2 complexes are unlikely to be responsible and that a more complex integration of signals is more likely involved.

**Conclusions:**

These findings suggest that proteolytic damage at sites of tissue injury and inflammation has the potential to modulate the VEGF system through a complex process and highlight the need for quantitative analysis to reveal mechanisms of growth factor control.

## Background

Vascular endothelial growth factor-A (VEGF) plays critical roles in vasculogenesis, angiogenesis and in maintaining vascular homeostasis [1,2]. VEGF functions as a mitogenic, chemotactic, and survival factor for endothelial cells, and has been shown to produce a pronounced angiogenic response in a variety of *in vivo *and *in vitro *models [1,2]. VEGF also induces vascular permeability and vasodilatation, as well as activities associated with non-vascular cell targets such as hematopoietic stem cells, monocytes, osteoblasts and neurons. Consistent with this wide range of important functions, deletion of even one allele of the VEGF-A gene is embryonic lethal showing impaired vasculogenesis and blood island formation [3,4]. Targeted inactivation of the VEGF-A gene in mouse lung causes an emphysematic phenotype suggesting that VEGF also plays critical roles in alveolar maintenance [5]. As a result of these critical activities, considerable attention has been paid to VEGF as a therapeutic agent and target for disease treatment. Focus has primarily been on heparin-binding VEGF_165_. However, direct VEGF delivery or inactivation using blocking antibodies produces mixed outcomes indicating that the natural mechanisms of VEGF control are complex and need to be better understood in order to design more effective VEGF/anti-VEGF therapies [2].

VEGF activity is principally mediated by VEGF receptors (VEGFR) 1 and 2 on vascular endothelial cells [1]. Interactions and signaling through these receptors are also modulated by the co-receptors neuropilin (NP) 1 and 2 [6,7]. VEGFR2 (also known as KDR and Flk-1) is a single pass transmembrane protein with high affinity for VEGF that is believed to be the major signaling receptor mediating the angiogenic activities of VEGF [1]. The importance of VEGFR2 is revealed by a lack of vasculogenesis and failure to develop blood islands and organized blood vessels in VEGFR2-null mice resulting in death *in utero *[8]. VEGFR1, on the other hand, has a higher affinity for VEGF but is a much less active tyrosine kinase. Hence, VEGFR1 is often thought to act as a 'decoy' receptor which functions by sequestering VEGF from its signaling receptors, VEGFR2 [9]. However, the wide expression of VEGFR1 on non-endothelial cells that do not express VEGFR2 suggests functions for VEGFR1 that are independent of this proposed 'decoy' role. NPs are cell surface glycoproteins that are proposed to function by presenting VEGF_165 _to VEGFR2. There is no evidence that NPs directly convey signals in response to VEGF binding, but these co-receptors appear necessary for VEGF-dependent angiogenesis [6] and some signal generation [7]. Interestingly, NP-1 has been shown to form direct complexes with VEGFR1 that result in decreased VEGF binding to NP-1 and VEGFR1, but the function of this interaction remains unclear [10]. Together these studies suggest that the relative levels of available VEGFRs and NPs on the cell surface may ultimately dictate VEGF response. This system is likely also influenced by the nature of the VEGF present, as some VEGF isoforms are unable to bind to NP-1 or show reduced affinity as does VEGF_121_[11].

**Figure 1:**
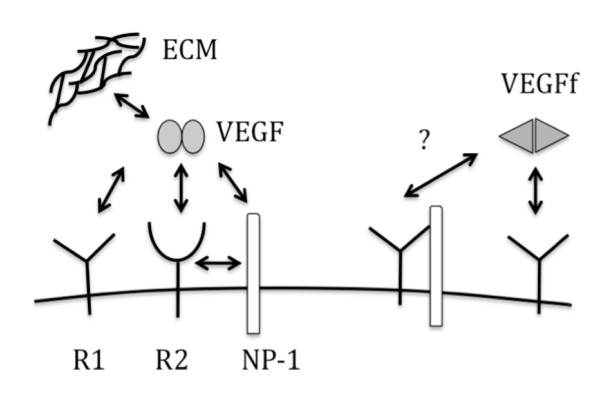
**Model illustrating VEGF and VEGFf binding interactions**. The following abbreviations are used: R1 for VEGFR1, R2 for VEGFR2, NP-1 for neuropilin-1. Question mark indicates that it is unknown if the binding reaction occurs.

VEGF is clearly an important regulatory protein subject to multiple levels of control. As noted above, expression of VEGF receptors and co-receptors can modulate function as can the expression of VEGF itself. In addition, the ability of VEGF to bind to components of the extracellular matrix, mainly heparan sulfate and fibronectin, has been suggested to control access of VEGF to cell surface receptors and provide a means for establishing gradients of VEGF for directed angiogenesis [12,13]. In this regard, we have recently reported that neutrophil elastase (NE) releases VEGF from the extracellular matrix (ECM) by processing it to a smaller form that shows altered binding and activity [14]. In particular, the NE-generated VEGF fragment (VEGFf) is unable to bind to VEGFR2 and shows reduced binding to heparin. It is possible that inflammatory proteases modulate VEGF receptor binding dynamics through the generation of VEGFf. Thus, in the present study, we have characterized the relative binding properties of VEGFf to NP-1, ECM and fibronectin and incorporated this information into a model of VEGF/VEGFf cell surface binding (Figure 1) to reveal possible mechanisms for VEGF activity control. These findings suggest that the generation of VEGFf by NE at sites of inflammation may modulate VEGF-mediated tissue repair by controlling cell surface binding events. This study highlights the complexity of the VEGF-VEGFR system and reveals the importance of applying system-wide analysis to identify non-intuitive insight into the mechanisms controlling ligand-receptor activation.

## Methods

### Materials

Human recombinant VEGF_165_, recombinant human Fc-VEGFR2 chimera, Fc-VEGFR1 chimera and Fc-Neuropilin 1 chimera were purchased from R&D systems (Minneapolis, MN). ^125^I-Bolton Hunter reagent was obtained from PerkinElmer (Boston, MA). Heparin was from Neoparin (San Leonardo, CA). Bovine serum albumin (BSA) was obtained from American Bioanalytical (Natick, MA). Purified human plasma fibronectin was from Chemicon International (Temecula, CA). Dulbecco's Modified Eagle's media (DMEM), phosphate buffered saline (PBS) containing no Ca_2+ _and Mg_2+_, penicillin/streptomycin, L-glutamine, N-(2-hydroxyethyl)piperazine-N'-2-ethanesulfonic acid (HEPES) and trypsin-EDTA were obtained from Invitrogen (Carlsbad, CA). Calf serum (CS) was purchased from Hyclone (Logan, UT). NE (human) was obtained from Elastin Products (Owensville, MO).

### Cell Culture

Bovine aortic endothelial cells (BAEC passage 5-15) were maintained in low-glucose DMEM, supplemented with 10% CS, 5 mM L-glutamine, 100 U/ml penicillin G, and 100 (μg/ml streptomycin sulfate. Neonatal rat aortic smooth muscle cells (SMCs) were isolated from Sprague-Dawley rats, ages 1-3 days as described, and maintained in low-glucose DMEM, with 10% FBS, 5 mM L-glutamine, 100 U/ml penicillin G, and 100 (μg/ml streptomycin sulfate and 1% nonessential amino acids.

### VEGF Fragment (VEGFf) Generation

^125^I-labeled VEGF_165_, prepared using a modified Bolton-Hunter procedure, was treated with varying NE concentrations in 44 mM sodium bicarbonate, pH 7.4, for various times at 37°C. Mock-treated ^125^I-VEGF was incubated in 44 mM sodium bicarbonate, pH 7.4 at 37°C. The reaction was stopped by adding 1 μM di-isopropyl fluorophosphate (DFP). For large VEGFf preparations, the samples (NE and mock treated) were dialyzed (10-kDa molecular weight cut off, Slide-A-Lyzer; Pierce, Rockford, IL) against PBS at 4°C to remove DFP. The concentration of ^125^I-VEGF_165 _and ^125^I-VEGFf in the samples was determined by trichloracetic acid precipitation.

### VEGF/VEGFf Binding to VEGFR1 and Neuropillin 1/Fc chimera

Binding assays were performed with VEGF binding chimeras by incubating a range of ^125^I-VEGF and ^125^I-VEGFf concentrations with Fc-VEGFR1, Fc-NP-1, or both in binding buffer (25 mM HEPES, pH 7.5, 150 mM NaCl, and 1 mg/ml BSA) for 2 h at 4°C. The bound complexes were pulled down with magnetic protein-A beads (New England Biolabs, Beverly, MA). The beads were washed three times with binding buffer, and ^125^I-VEGF/^125^I-VEGFf associated with the beads was measured using a Cobra Auto-Gamma 5005 counter (Packard Instruments, Meridian,CT).

### VEGF/VEGFf Binding to Fibronectin

Fibronectin (10 μg/ml) in the presence of heparin (10 μg/ml) was adsorbed overnight onto 96-well hydrophobic polystyrene plates in PBS at 4°C (100 μl/well). Following protein adsorption, the surface was washed three times with PBS and once with binding buffer. VEGF/VEGFf binding assays were then conducted with various concentrations of ^125^I-VEGF and ^125^I-VEGFf in 0.15 M NaCl, 25 mM HEPES, 1 mg/ml BSA, pH 7.5 for 2.5 h at 4°C (50 μl/well). In some cases, plates were treated with various NE concentrations for 30 min at 37°C, the NE solution removed and plates washed three times with PBS then incubated for 10 min with PBS containing 1 μM DFP to inhibit any residual NE. VEGF binding to treated plates was then measured by incubating the wells with the indicated concentration of ^125^I-VEGF/VEGFf. Unbound VEGF/VEGFf was removed by washing three times with binding buffer and bound VEGF/VEGFf was extracted by 1 h incubation with 5M NaCl, 25 mM HEPES, pH 7.5. The incubation was performed at room temperature with 50 μl of extraction solution per well. After each incubation period the wells were washed once more with the same extraction solution (50 μl/well) to ensure complete recovery of the respective fraction of bound VEGF/VEGFf. The radioactivity released was measured by a Cobra Auto-Gamma 5005 counter (Packard Instruments, Meridian, CT).

### VEGF Binding to Cell Culture Extracellular Matrices

Primary rat aortic smooth muscle cells and pulmonary fibroblasts were plated onto 24-well plates (2 cm^2^/well) at an initial density of 5 × 10^4 ^per well. When the cells reached confluence, the culture media was changed to 1% serum. Five days past-confluence ^125^I-VEGF/VEGFf binding was conducted with isolated ECM. Isolated ECM was used to evaluate the effects of elastase on VEGF binding in order to avoid interference caused by the effects of high elastase concentrations on the living cells. To prepare cell-free ECM the cell layer was dissolved with 0.5% Triton, 20 mM NH_4_OH in PBS at 23°C for 3 min, followed by three washes with PBS. The isolated ECM was incubated with various concentrations of NE for 30 min at 37°C, the elastase was inactivated with 1 μM DFP and the ECM plates washed with PBS. Isolated ECM were incubated with ^125^I-VEGF or ^125^I-VEGFf in binding buffer (0.15 M NaCl, 25 mM HEPES, pH 7.5, 1 mg/ml BSA) at 4°C for 2 h. Unbound ligand was removed by washing the ECM layers three times with binding buffer and VEGF/VEGFf bound to ECM sites was extracted with 2 M NaCl, 25 mM HEPES, pH 7.5 and samples were counted in a gamma counter.

### Cell Migration Assay

Migration assays were performed using a modified Boyden chamber technique using 24-well Transwell^® ^permeable supports (Corning, NY) with migration inserts (5 μm pore size, 6.5 mm diameter). Serum starved BAEC were plated onto the transwell inserts at a density of 100,000 cells/insert in serum-free medium, 25 mM HEPES, pH 7.5 and 0.05% gelatin (v/v) and placed in 24-well plates containing binding buffer +/- chemoattractant (VEGF, VEGFf) at 0.45 nM. The assembled plate was placed at 37°C (5% CO_2_, humidified) for the duration of the migration time (2h). Once the migration time had finished, media from the lower and upper chambers were aspirated; migrated cells were washed once in PBS without Ca^2+ ^and Mg^2+ ^ions and the cells that had migrated to the other side of the membrane were fixed with 100% pre chilled methanol for 10 min. Cells were subsequently washed two times in PBS and the non migrated cells on the top side of the transwell membrane were swabbed using Q-tips. The migrated cells were then stained by incubating in 5 μg/ml propidium iodide in PBS (600 μl/well) for 10 min. Cells were subsequently washed two times in PBS and a microsurgery knife was used to cut out the transwell membrane. The membranes were placed on labeled glass slides with migrated cells facing up. A drop of Antifade Component A (Molecular Probes, Invitrogen, Carlbad, CA) was placed on top of each membrane prior to covering with a glass cover slip. Images of migrated cells were captured by fluorescent microscopy at six different fields/membrane at 100× magnification. The migrated cells were counted using Image J NIH software.

### Statistics

Experimental data was subjected to statistical analysis using the Analysis ToolPak in Microsoft Excel X for Mac. Dose response data were subject to regression analysis and analysis of variance (ANOVA). Data from multiple treatments/conditions was subjected to ANOVA followed by the Newman-Keuls multiple comparison t-test routine. Differences between groups were considered statistically significant when *p *was less than 0.05.

### Computational Model Development

In this paper we develop a model of VEGF binding and regulation of cell surface binding by VEGFf (Figure 1, Table 1). The model is based on mass-action kinetics describing binding and cell surface trafficking interactions for VEGF with VEGFR1, VEGFR2, NP-1, and ECM sites and builds on previous work from Popel and co-workers[15-20]. In the model, VEGF can form a triad with VEGFR2 and NP-1 but not with VEGFR1. NP-1 can bind VEGFR1 in the absence of VEGF but not VEGFR2. Coupling and uncoupling rates were considered equivalent for all species and independent of VEGF binding. A 1:1 stoichiometry of VEGF to receptor or NP-1 was maintained. Heparan sulfate proteoglycans (HSPGs) were only explicitly included as ECM sites. Synthesis was based on steady-state receptor levels and internalization rates for all species were considered equivalent except where noted. The fluid phase is considered well-stirred.

**Table 1:** Model Reactions

VEGF	VEGFf
*R*_*1 *_*+ V *↔ *C*_*R1*_	Model 1&2
*R*_*2 *_+ *V *↔ *C*_*R2*_	*R*_1 _+ *F *↔ *D*
*E + V *↔ *C*_*E*_	Model 1 only
*N + V *↔ *C*_*N*_	*D + N *↔ *T*_*F*_
*C*_*R2 *_+ *N *↔ *T*_*V*_	*X + F *↔ *T*_*F*_
*C*_*N *_+ *R*_*2 *_↔ *T*_*V*_	
*R*_*1 *_+ *N *↔ *X*	

V = VEGF, F = VEGFf, R_1 _= VEGFR1, R_2 _= VEGFR2, N = NP-1, E = ECM, C_R1 _= VEGF-VEGFR1, C_R2 _= VEGF-VEGFR2, C_E _= VEGF-ECM, C_N _= VEGF-NP-1, D = VEGFf-VEGFR1, T_V _= VEGF-VEGFR2-NP-1,T_F _= VEGFf-VEGFR1-NP-1, X = VEGFR1-NP-1

Regulation of VEGF binding by VEGFf was investigated in two ways. VEGFf has been shown previously to bind to VEGFR1 with a similar affinity as VEGF, but does not bind VEGFR2[14]. In this paper, we provide evidence that VEGFf does not bind to NP-1 or ECM sites. The model reflected these events but there is no direct evidence supporting or excluding VEGFf bound to VEGFR1 from interacting with NP-1 as shown for VEGF_121_[10] but not VEGF. In model 1, VEGFf was therefore able to bind VEGFR1 and then couple with NP-1 or bind to VEGFR1-NP-1 complexes. In model 2, binding of VEGFf to VEGFR1 excluded further interactions with NP-1 and VEGFf was not able to bind to VEGFR-NP-1 complexes. In all other ways the models are identical.

The models are composed of a set of nonlinear ordinary differential equations. The equations listed below describe Model 2, where VEGFf bound to VEGFR1 excludes any interactions between VEGFR1 and NP-1. Symbols are defined in Tables 1 and 2.

(1) $$\frac{dC_{R1}}{dt}=k_{f}^{VR1}VR_{1}-k_{r}^{VR1}C_{R1}-k_{\text{int}\;}^{C}C_{R1}$$

(2) $$\begin{array}{c} \frac{dC_{R2}}{dt}=k_{f}^{VR2}VR_{2}-k_{r}^{VR2}C_{R2}\\ -k_{c}^{C2N}C_{R2}N+k_{uc}^{C2N}T_{V}-k_{\text{int}\;}^{C}C_{R2} \end{array}$$

(3) $$\frac{dC_{E}}{dt}=k_{f}^{VE}VE-k_{r}^{VE}C_{E}$$

(4) $$\begin{array}{c} \frac{dC_{N}}{dt}=k_{f}^{VN}VN-k_{r}^{VN}C_{N}-k_{c}^{CNR2}C_{N}R_{2}\\ +k_{uc}^{CNR2}T_{V}-k_{\text{int}\;}^{C}C_{N} \end{array}$$

(5) $$\frac{dX}{dt}=k_{c}^{R1N}R_{1}N-k_{uc}^{R1N}X-k_{\text{int}\;}X$$

(6) $$\begin{array}{c} \frac{dR_{1}}{dt}=S_{R1}-k_{f}^{VR1}VR_{1}+k_{r}^{VR1}C_{R1}-k_{c}^{R1N}R_{1}N\\ +k_{uc}^{R1N}X-k_{f}^{FR1}FR_{1}+k_{r}^{FR1}D-k_{\text{int}\;}R_{1} \end{array}$$

(7) $$\begin{array}{c} \frac{dR_{2}}{dt}=S_{R2}-k_{f}^{VR2}VR_{2}+k_{r}^{VR2}C_{R2}-k_{c}^{CNR2}C_{N}R_{2}\\ +k_{uc}^{CNR2}T_{V}-k_{\text{int}\;}R_{2} \end{array}$$

(8) $$\frac{dE}{dt}=-k_{f}^{VE}VE+k_{r}^{VE}C_{E}$$

(9) $$\begin{array}{c} \frac{dN}{dt}=S_{N}-k_{f}^{VN}VN+k_{r}^{VN}C_{N}-k_{c}^{R1N}R_{1}N\\ +k_{uc}^{R1N}X-k_{f}^{C2N}C_{R2}N+k_{f}^{C2N}T_{V}-k_{\text{int}\;}N \end{array}$$

(10) $$\frac{dD}{dt}=k_{f}^{FR1}FR_{1}-k_{r}^{FR1}D-k_{\text{int}\;}^{F}D$$

(11) $$\begin{array}{c} \frac{dT_{v}}{dt}=k_{c}^{CNR2}C_{R2}N-k_{uc}^{CNR2}T_{V}+k_{c}^{C2N}C_{R2}N\\ -k_{uc}^{C2N}T_{V}-k_{\text{int}\;}^{C}T_{V} \end{array}$$

(12) $$\begin{array}{c} v\frac{dV}{dt}=-k_{f}^{VR1}VR_{1}+k_{r}^{VR1}C_{R1}-k_{f}^{VR2}VR_{2}+k_{r}^{VR2}C_{R2}\\ -k_{f}^{VN}VN+k_{r}^{VN}C_{N}-k_{f}^{VE}+k_{r}^{VE}C_{E} \end{array}$$

(13) $$v\frac{dF}{dt}=-k_{f}^{FR1}FR_{1}+k_{r}^{FR1}D$$

With Model 1 where VEGFf bound VEGFR1 can interact with NP-1 the following equations are altered:

(5b) $$\frac{dX}{dt}=k_{c}^{R1N}R_{1}N-k_{uc}^{R1N}X-k_{f}^{FX}FX+k_{r}^{FX}T_{F}-k_{\text{int}\;}X$$

(10b) $$\frac{dD}{dt}=k_{f}^{FR1}FR_{1}-k_{r}^{FR1}D-k_{c}^{D}DN+k_{uc}^{DN}T_{F}-k_{\text{int}\;}^{F}D$$

(13b) $$v\frac{dF}{dt}=-k_{f}^{FR1}FR_{1}+k_{r}^{FR1}D-k_{f}^{FX}FX+k_{r}^{FX}T_{F}$$

and added:

(14) $$\frac{dT_{F}}{dt}=k_{f}^{FX}FX-k_{r}^{FX}T_{F}+k_{c}^{DN}DN-k_{uc}^{DN}T_{F}-k_{\text{int}\;}^{F}T_{F}$$

Simulations were run in Matlab R2006b (The Mathworks, Inc., Natick, MA) using the stiff ordinary differential equation solver ode15s with the backwards differentiation formulas option and an absolute tolerance criteria of 1 × 10^-20^. Parameter values are listed in Table 2. Simulations were generally run for 3h. Values were used as reported in the literature with no attempt to adjust based on temperature.

**Table 2:** Model Parameters*

Symbol	Value	Meaning
R_1_(t = 0)	65,000 #/cell	# of VEGFR1 per cell at time zero
R_2_(t = 0)	12,000 #/cell	# of VEGFR2 per cell at time zero
N(t = 0)	10,000 #/cell	# of Neuropilin-1 per cell at time zero
E(t = 0)	1,000,000 #/cell	# of ECM sites per cell which can bind VEGF at time zero
$k_{f}^{VR1};k_{r}^{VR1}$	3 × 10^7 ^M^-1 ^s^-1^; 1 × 10^-3 ^s^-1^	Association/dissociation rate constant for VEGF binding to VEGFR1
$k_{f}^{VR2};k_{r}^{VR2}$	1 × 10^7 ^M^-1 ^s^-1^; 1 × 10^-3 ^s^-1^	Association/dissociation rate constant for VEGF binding to VEGFR2
$k_{f}^{VE};k_{r}^{VE}$	4.2 × 10^5 ^M^-1 ^s^-1^; 1 × 10^-2 ^s^-1^	Association/dissociation rate constant for VEGF binding to ECM (based on HS values from literature)
$k_{f}^{VN};k_{r}^{VN}$	3.125 × 10^6 ^M^-1 ^s^-1^; 1 × 10^-3 ^s^-1^	Association/dissociation rate constant for VEGF binding to NP-1
$k_{f}^{FR1};k_{r}^{FR1}$	3 × 10^7 ^M^-1 ^s^-1^; 1 × 10^-3 ^s^-1^	Association/dissociation rate constant for VEGFf binding to VEGFR1(modeled the same as VEGF)
$k_{c}^{C2N};k_{uc}^{C2N}$	3.1 × 10^13 ^(mol/cm^2^)^-1 ^s^-1^; 1 × 10^-3 ^s^-1^	Coupling/uncoupling rate constant for VEGF-VEGFR2 with NP-1
$k_{c}^{R1N};k_{uc}^{R1N}$	1 × 10^14 ^(mol/cm^2^)^-1 ^s^-1^; 1 × 10^-2 ^s^-1^	Coupling/uncoupling rate constant for VEGFR1 with NP-1
$k_{c}^{CNR2};k_{uc}^{CNR2}$	1 × 10^14 ^(mol/cm^2^)^-1 ^s^-1^; 1 × 10^-3 ^s^-1^	Coupling/uncoupling rate constant for VEGF-NP-1with VEGFR2
$k_{f}^{FX};k_{r}^{FX}$	$k_{f}^{FR1};k_{r}^{FR1}$	Association/dissociation rate constant for VEGFf binding to VEGFR1-NP-1
$k_{c}^{DN};k_{uc}^{DN}$	$k_{c}^{R1N};k_{uc}^{R1N}$	Coupling/uncoupling rate constant for VEGFf-VEGFR1 with NP-1
*k*_int_	2.8 × 10^-4 ^s^-1^	Internalization rate for unbound receptors
$k_{\text{int}\;}^{C}$	2.8 × 10^-4 ^s^-1^	Internalization rate for VEGF-bound receptors
$k_{\text{int}\;}^{F}$	2.8 × 10^-4 ^s^-1^	Internalization rate for VEGFf-bound receptors
*S*_I_	*k*_int_*I*_0_	Synthesis rate for species I where *I*_0 _is the initial concentration of I

*Values are from literature when possible [20]. VEGFf assumed to interact in similar manner to VEGF when applicable.

## Results

### Elastase Degrades VEGF Binding Sites in the ECM

**Figure 2:**
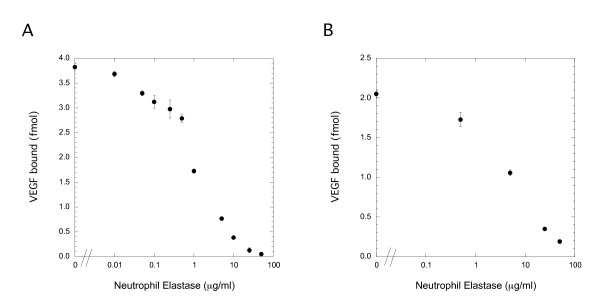
**Pre-treatment of fibronectin and SMC-derived ECM with NE decreases VEGF binding**. A: 96-well hydrophobic plates were coated with 10 μg/ml fibronectin in the presence of 10 μg/ml heparin. Plates were treated with NE then ^125^I-VEGF (0.45 nM) was added for 2 h. Bound VEGF was released with 2 M NaCl, 25 mM HEPES, pH 7.5. B: Primary rat smooth muscle cells were grown 5 days past confluence, cell layer dissolved, and the exposed extracellular matrix was incubated with NE. ECM coated wells were then incubated with ^125^I-VEGF (0.45 nM) and bound VEGF extracted. Each data point is the mean of quadruplicate determinations ± SEM. Regression analyses with ANOVA of both dose response curves revealed a statistically significant effect of NE treatment with *Significance F *and *p *values below 0.05.

Elastase is known to degrade ECM components including HSPGs [21-23], thus, we explored the possibility that elastase degrades VEGF binding sites within the ECM. To do this, we isolated ECM from rat smooth muscle cell cultures, subjected these ECM preparations to elastase digestion and then measured VEGF binding. Previous studies have indicated that fibronectin is a major VEGF binding site in the ECM. Thus, we also investigated the possibility that elastase destroys VEGF binding sites on fibronectin using polystyrene surfaces coated with fibronectin. Elastase treatment of smooth muscle cell-deposited ECM or fibronectin matrices resulted in a dramatic loss of VEGF binding capacity (Figure 2). Approximately 50% loss in binding was observed with 5 and 1 μg/ml elastase treatments of ECM and fibronectin respectively, while high doses of elastase (50 μg/ml) resulted in >90% loss of binding to either matrix. Therefore, elastase treatment at concentrations that have previously been shown to cause growth factor release from ECM [21-23] would be predicted to cause a loss of VEGF binding sites within the ECM.

### Simulations Indicate ECM Sites Have Minimal Impact on VEGF binding to Cell Surface Receptor

**Figure 3:**
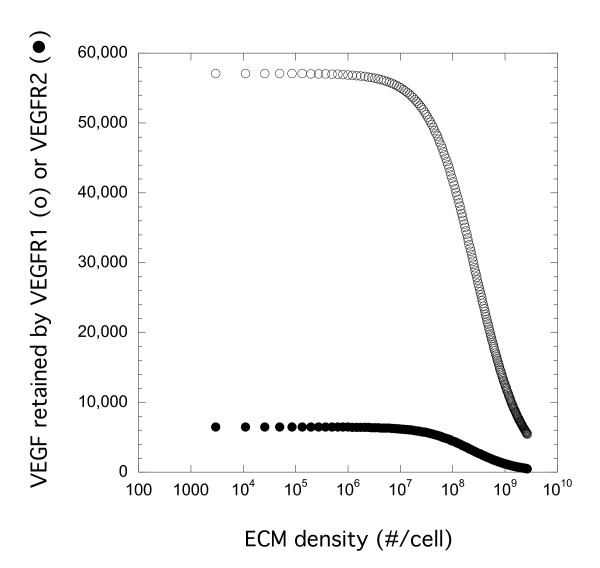
**ECM Density Impacts Receptor Binding of VEGF at High Densities**. Simulation results following introduction of VEGF (0.023 nM or 1 ng/ml) at time zero. VEGF retained by VEGFR1 (open) or VEGFR2 (filled) after 3 h of VEGF exposure is shown. All other parameter values were at base values (Table 2) except for ECM site density.

ECM sites for VEGF are generally numerous but of low-affinity [15,24]. Using our VEGF model (Table 1, described in Methods), we investigated how the density of ECM sites impacted VEGF receptor binding using simulations. VEGF (0.023 nM) was added at time zero and the levels of VEGF-VEGFR1 and VEGF-VEGFR2 complexes were determined after three hours of simulation time (Figure 3). Under these conditions (Table 2), there was considerable retention (surface and internalized complexes) of VEGF to both VEGFR1 and VEGFR2 with over five times as many VEGFR1 complexes as VEGFR2 complexes across the range of ECM density investigated. Similar results were found when we looked at just surface complexes at 3 h instead of total complexes (surface and internalized) or when internalization and synthesis were eliminated in simulations of 4°C binding data (data not shown). These different binding levels were a consequence of the higher density of VEGFR1 and the higher binding affinity of VEGF for this receptor (Table 2). The density of ECM sites had a negligible effect on VEGF binding to either VEGFR1 or VEGFR2 except at densities greater than 10^7 ^sites/cell where inhibition becomes significant. This is due to the low affinity of these sites for VEGF compared to VEGFR1 or VEGFR2. If the affinity of the ECM site is significantly higher, the effect would be greater (data not shown). At typical cell densities measured in cultures (10^6 ^sites/cell and lower) and affinities, degradation of ECM sites would not be predicted to have a significant effect on receptor binding of VEGF and would therefore be unlikely to impact cell activity.

### VEGF fragment Exhibits Negligible Binding to ECM, Fibronectin and Neuropilin-1

**Figure 4:**
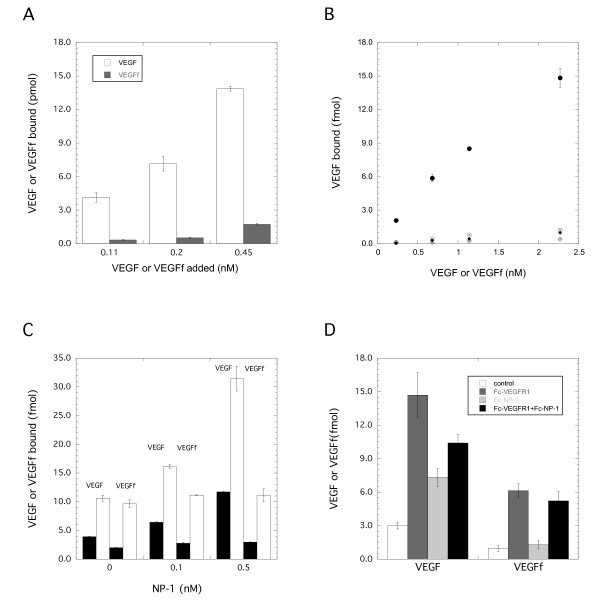
**VEGF and VEGFf binding interactions. A: VEGF/VEGFf binding to ECM from pulmonary fibroblasts**. ^125^I -VEGF (open) or ^125^I -VEGFf (filled) was incubated for 3 hours at 4°C and bound growth factor was determined. Significant differences between VEGF and VEGFf binding was found. B: VEGF/VEGFf binding to fibronectin ± heparin. ^125^I-VEGF (circles) or ^125^I-VEGFf (diamonds) bound to fibronectin alone (open) or with heparin (filled). C: VEGF/VEGFf binding to NP-1. ^125^I -VEGF or ^125^I -VEGFf at 0.25 (filled) and 1 (open) nM were incubated with Fc-NP-1 for 2 h and complexes determined. Significant differences were found between VEGF bound in the presence of 0.5 nM NP-1 compared to no NP-1, and with 0.25 nM VEGF in the presence of 0.1 nM NP-1 compared to no NP-1. No significant differences were noted for VEGFf ± NP-1. D: VEGF/VEGFf binding to VEGFR1 and NP-1. 0.25 nM ^125^I -VEGF or ^125^I -VEGFf was incubated alone (white) or with Fc-VEGFR1 (0.1 nM, dark grey), Fc-NP-1 (0.5 nM, light grey), or both VEGFR1 and NP-1 (black) for 2 h; complexes were pulled down and ^125^I-VEGF/VEGFf counted. Significant differences were observed with all VEGF bound groups, and between VEGFf bound in the presence versus the absence of VEGFR1. No significant difference was noted between VEGFf bound to VEGFR1 ± NP-1. Data are the mean of quadruplicate (A,C) triplicate (D) or duplicate (B) determinations ± SD. ANOVA followed by multi-comparison t-tests was performed for statistical analysis.

We have shown, however, that the impact of elastase with regard to the VEGF system is not limited to the ECM binding sites. Elastase cleaves VEGF to produce a smaller fragment of VEGF (VEGFf) with cleavage sites at the C-terminal, N-terminal and internal regions that exhibits reduced binding to heparin and VEGFR2 but retains the ability to bind VEGFR1[14]. We therefore tested whether VEGFf bound to cell-deposited ECM. VEGF bound ECM in a dose-dependent manner (Figure 4A) while VEGFf exhibited negligible binding indicating that the conversion to VEGFf would likely impact ECM storage of the growth factor.

In related studies, we found that heparin exposure significantly increases VEGF binding to fibronectin catalyzing the conversion of fibronectin to an open conformation [24,25]. Thus, to more fully characterize the effects of elastase on VEGF activity, we prepared VEGFf and assessed its binding to fibronectin ± heparin. Fibronectin ± heparin was adsorbed on hydrophobic polystyrene surfaces and ^125^I-VEGF and ^125^I-VEGFf binding measured. Intact VEGF showed minimal binding to fibronectin in the absence of heparin exposure but showed significantly enhanced binding to fibronectin that was previously incubated with heparin (Figure 4B). This heparin-enhanced VEGF binding was 5 to 14 fold higher compared to VEGF binding to fibronectin alone over the range of VEGF concentrations tested. In contrast, VEGFf showed no significant binding to fibronectin in the presence or absence of heparin. The lack of VEGFf binding to fibronectin is consistent with an overall reduced ability to bind to ECM binding sites as compared to intact VEGF.

NP-1 is a co-receptor for VEGF that enhances angiogenic signaling cooperatively with VEGFR2. Therefore we examined whether VEGFf was able to bind to NP-1 and if this interaction would influence interactions with VEGFR1. To do so, we performed cell-free receptor binding experiments using Fc-NP-1 and Fc-VEGFR1 chimeric proteins. The chimeras were composed of the extracellular VEGF binding domain of the relevant receptor fused to the Fc-region of human IgG_1 _via a peptide linker. ^125^I-VEGF or ^125^I-VEGFf was incubated with Fc-NP-1, Fc-VEGFR1 or both for 2h. Bound complexes were measured after pull down with magnetic protein A beads. VEGFf showed no significant binding to Fc-NP-1 chimera at either concentration tested (0.25 or 1 nM) while intact VEGF bound Fc-NP-1 (Figure 4C) indicating that elastase processing of VEGF alters its ability to bind NP-1. Previous studies have reported that NP-1 is able to inhibit VEGF binding to VEGFR1 by potentially heterodimerizing with VEGFR1 and preventing VEGF-VEGFR1 binding. To test whether VEGFf binding to VEGFR1 is affected by the presence of NP-1, Fc-VEGFR1 chimeras were pre-incubated with NP-1 prior to evaluating VEGF/VEGFf binding. Intact VEGF bound to both Fc-VEGFR1 and Fc-NP-1 but the combination value was below that for Fc-VEGFR1 alone indicating no additive effect for the combination and suggesting that VEGFR1 interactions with VEGF were reduced by the presence of NP-1 (Figure 4D). VEGFf bound to Fc-VEGFR1 but not Fc-NP-1 and the addition of Fc-NP-1 had a negligible impact on VEGFf overall binding. This suggests that either (1) VEGFf binding to VEGFR1 is unaffected by NP-1 and that there is no difference in VEGFf affinity for VEGFR1 alone or VEGFR1-bound to NP-1 or that (2) VEGFf binding to Fc-VEGFR1 inhibits VEGFR1 binding to NP-1 and that the VEGFf interaction is of a higher affinity than the VEGFR1-NP-1 interaction. In either case, these results suggest that the inability of VEGFf to bind NP-1 is reflective of a general loss of NP-1 control over the system. We decided to investigate how these two possibilities for VEGFf interactions with VEGFR1 and NP-1 would be predicted to impact the VEGF system using computational modeling.

### Computational Model Shows that VEGF Fragment Impacts VEGF-VEGFR2 Complex Levels Despite Not Binding to VEGFR2

A computational model including VEGF, VEGFR1, VEGFR2, NP-1 and VEGFf was developed. In our model, VEGFf is able to bind VEGFR1 with the same affinity as does VEGF but it is not able to bind either VEGFR2 or NP-1. Our experimental results, however, were unclear regarding VEGFf interactions with VEGFR1-NP-1 complexes and so we investigated this question using two variations of the model. In Model 1, VEGFf can bind to VEGFR1-NP-1 complexes and VEGFf bound to VEGFR1 can bind to NP-1, similar to VEGF_121_[10]. In Model 2, VEGFf binding to VEGFR1 excludes interactions between VEGFR1 and NP-1 entirely. With Model 2, there are no VEGFf-VEGFR1-NP-1 complexes. In the simulations, VEGF (1 ng/ml; 0.023 nM) and VEGFf were both added at time zero and simulations run corresponding to three hours of experimental incubation. These conditions might correspond to the case where VEGFf has been generated from matrix-bound VEGF by elastase and the VEGFf is now soluble and capable of impacting VEGF binding downstream.

**Figure 5:**
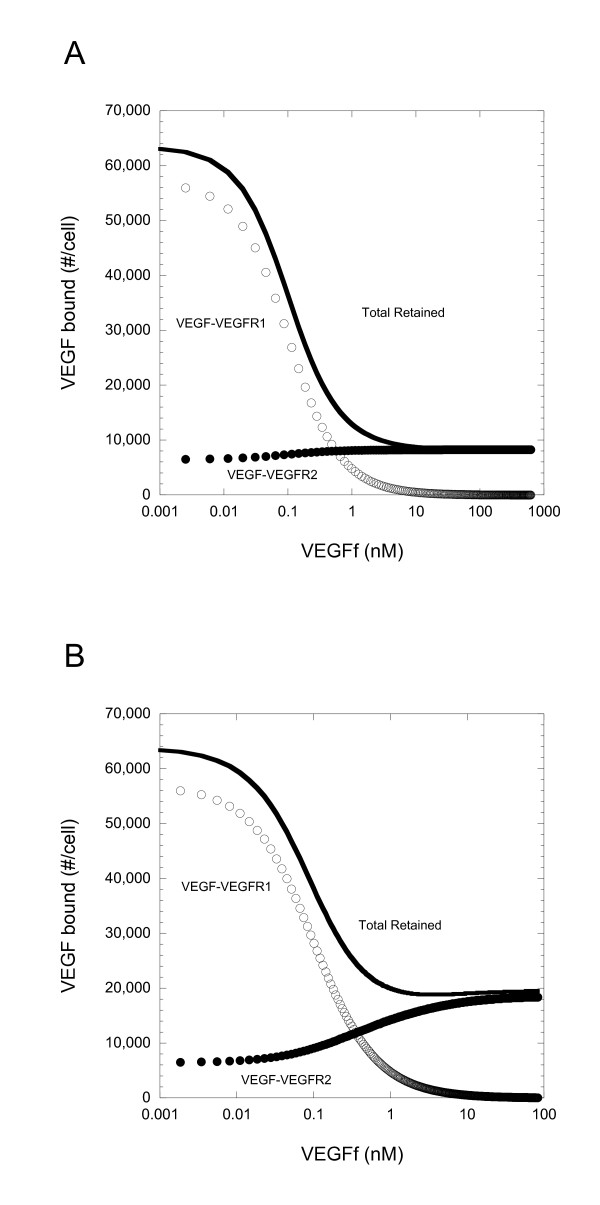
**Impact of VEGFf on VEGF-Receptor binding differs when VEGFf interacts with VEGFR1 and NP-1**. Simulation results for (A) Model 1 (VEGFf can bind to VEGFR1 and NP-1) and (B) Model 2 (VEGFf cannot bind to VEGFR1 and NP-1) 3 h after introduction of VEGF(1 ng/ml or 0.023 nM) and VEGFf to the cell system. VEGF bound to VEGFR1 (open), VEGFR2 (filled), and sum of VEGF bound to VEGFR1, VEGFR2, NRP, and ECM (solid line)

Using our model, in the absence of VEGFf, there was greater than five times more VEGF-VEGFR1 complexes than VEGF-VEGFR2 complexes due to the increased affinity and density of VEGFR1 (Figure 5). The addition of VEGFf reduced VEGF-VEGFR1 complexes with both Model 1 and Model 2 to a similar extent. VEGFf competes directly with VEGF for binding to VEGFR1 and so increasing the concentration of VEGFf resulted in an expected reduction in VEGF-VEGFR1 complexes. In contrast, the effect of VEGFf on VEGF binding to VEGFR2 was markedly different depending on whether VEGFf binding to VEGFR1 excluded interactions with NP-1.

With Model 1 (Figure 5A), where NP-1 can interact with VEGFf-VEGFR1 complexes, even the addition of high concentrations of VEGFf had only a small but positive effect (< 2%) on the level of VEGF retained by VEGFR2 (bound + internalized) (Figure 5A). In contrast, when VEGFf binding inhibits interactions between VEGFR1 and NP-1 (Model 2), addition of fragment more than doubled the level of VEGF retained by VEGFR2 (Figure 5B). The total amount of VEGF retained by the cells in the presence of VEGFf reflects these differences in VEGFR2 binding with Model 1 showing only inhibition while Model 2 showed a biphasic response. Similar relative results were found when looking at surface bound only (excluding internalized complexes) or when synthesis and internalization were excluded from the model (results not shown).

### Neuropilin Sequestration Impacts VEGF-VEGFR2 Complex Levels

**Figure 6:**
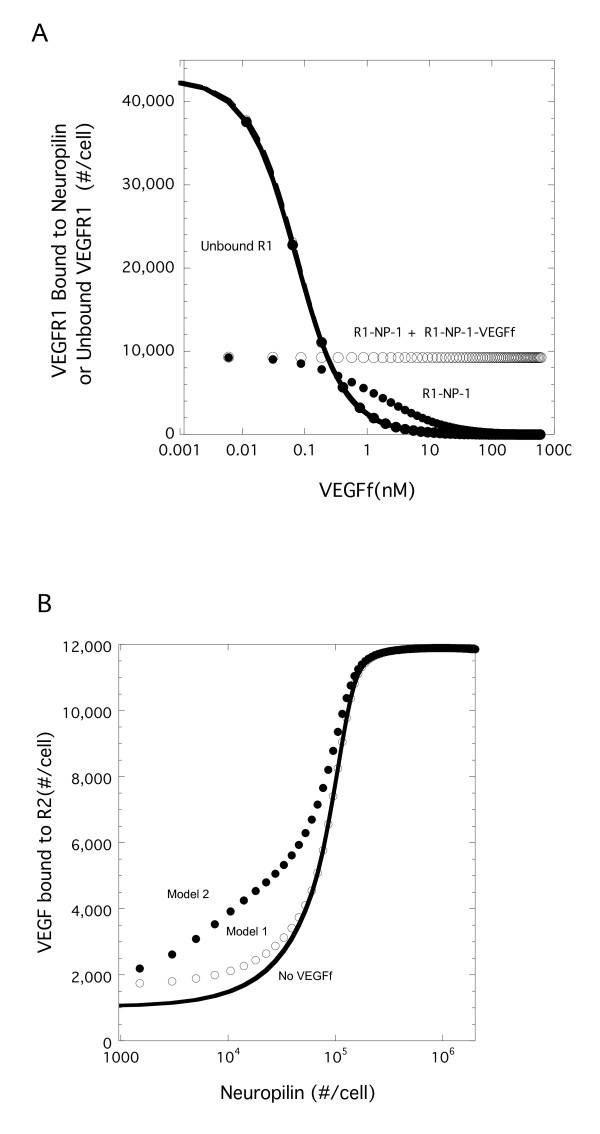
**NP-1 sequestering by VEGFR1 is key to difference in VEGF binding to VEGFR2**. (A) Simulations were run with VEGF (0.023 nM) and variable VEGFf for 3 h using standard parameter values (Table 2). VEGFR1 bound to NP-1 or unbound VEGFR1 are shown for Model 1 (VEGFf can bind to VEGFR1 and NP-1 - open) and Model 2 (VEGFf cannot bind to VEGFR1 and NP-1 -filled). Note the direct overlap of the unbound VEGFR1 for the two models. (B) Similar simulations were run but with 0 (solid line) or 1 nM VEGFf for Model 1 (open) and Model 2 (filled) with variable levels of NP-1. VEGF bound to VEGFR2 is shown.

The difference in VEGF-VEGFR2 complexes in response to VEGFf between the two models was striking. When we looked at the level of VEGFR1 that was not bound to VEGF, VEGFf or NP-1, we found that these levels decreased with increasing VEGFf until there were essentially no free VEGFR1 with either Model (Figure 6A). In contrast, VEGFR1 bound to NP-1 was not significantly altered by the presence of VEGFf with Model 1, yet was dramatically reduced with Model 2. This reduction in VEGFR1-NP-1 complexes in Model 2, as a result of the binding of VEGFf to VEGFR1 and the subsequent exclusion of NP-1 from the VEGFR1-NP-1 complex, leads to an increase in NP-1 available for VEGF-VEGFR2 stabilization. This stabilization leads to increased VEGFR2 complexes and resulting increased total retention of VEGF found with Model 2, as the increase VEGFR2 binding is greater than the loss of VEGFR1 binding in these simulations (Figure 5B).

NP-1 levels had a critical impact on VEGF retention by VEGFR2 (Figure 6B). Despite having an equivalent level of VEGF, higher levels of NP-1 resulted in near saturation of binding and a more than 5 fold increase in binding levels over that at the lowest level of NP-1. Model 2 enhances VEGF-VEGFR2 levels over the lower range of NP-1 values investigated compared to both VEGF-VEGFR2 levels with Model 1 as well as to the situation when VEGF-VEGFR2 binding occurs in the absence of VEGFf (control case). Note that VEGF-VEGFR2 retention in the absence of VEGFf (control case) is the same for Model 1 and Model 2 since the difference between the models lies only in the binding of VEGFf.

### Coupling Between VEGFR1 and NP-1 Regulates VEGF-VEGFR2 Binding

**Figure 7:**
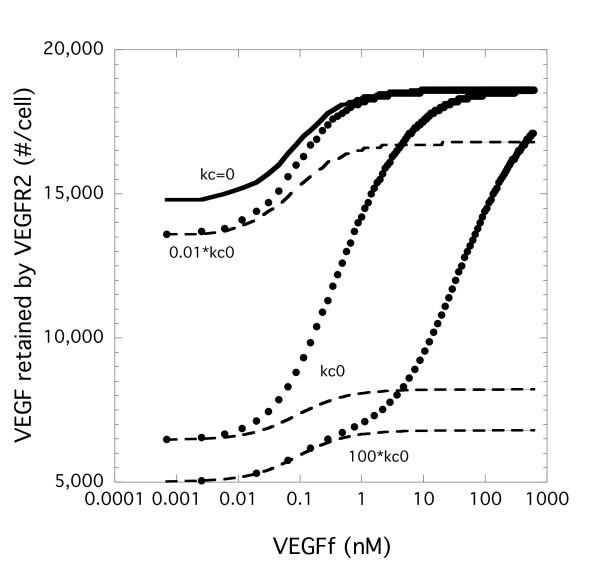
**Coupling rate constant between VEGFR1 and NP-1 impacts VEGF binding to VEGFR2**. Simulations were performed with Model 1 (VEGFf can bind to VEGFR1 and NP-1 - dashed line) and Model 2 (VEGFf cannot bind to VEGFR1 and NP-1 - filled circles) with VEGF (0.023 nM) and variable VEGFf for 3 h using base parameter values except for the coupling rate between VEGFR1 and NP-1. Total VEGFR2 bound to VEGF (surface and internal) is shown. kc0 is the base value shown in Table 2. kc = 0 is equivalent for both models and is shown in the solid line.

The importance of the NP-1 levels as well as the differences observed with the two VEGFf binding models suggest that coupling between VEGFR1 and NP-1 is critical to regulation of VEGF-VEGFR2 binding both in the presence or absence of VEGFf. In the absence of coupling between the two surface proteins (VEGFR1 and NP-1), VEGFf had an impact (~15%) on VEGF retention by VEGFR2 (Figure 7, kc = 0). This impact was significantly decreased (~3%) when endocytosis/synthesis was not included (data not shown). At our baseline coupling value (kc0, Table 2), VEGF binding to VEGFR2 was decreased compared to that found in the absence of coupling (kc = 0) for both models, but it increased by approximately 3-fold when VEGFf was added for Model 2 (large filled dots). At the highest VEGFf value, the amount of VEGF retained by VEGFR2 is the same as that found in the absence of coupling (kc = 0) for Model 2. Decreasing the coupling value increases the percentage of VEGF-bound VEGFR2 while increasing the coupling value had the opposite effect. VEGFf had a smaller impact on the retention of VEGF by VEGFR2 with Model 1 irrespective of the coupling rate (dotted lines in Figure 7). This impact was negligible when endocytosis/synthesis was not included (data not shown). Looking only at surface bound VEGF-VEGFR2 showed similar trends to that shown in Figure 7 (data not shown).

### Relative Receptor Levels and Receptor-Mediated Endocytosis Impact VEGF Capture

**Figure 8:**
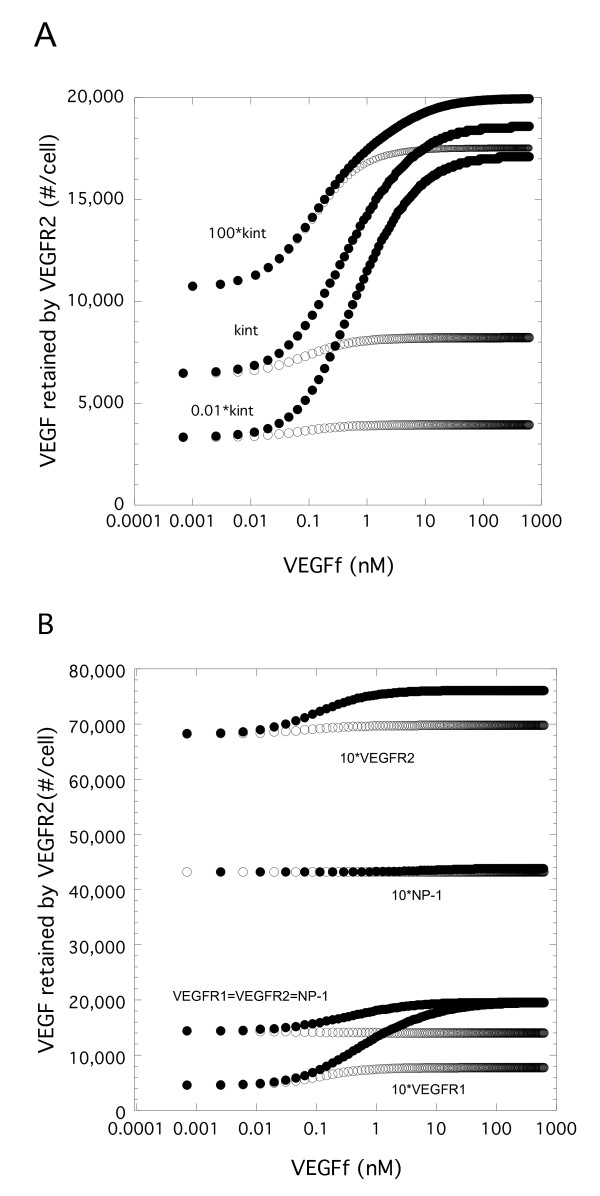
**VEGF-mediated endocytosis and relative receptor levels impacts VEGF binding to VEGFR2**. A. Simulations were performed for Model 1 (VEGFf can bind to VEGFR1 and NP-1) and Model 2 (VEGFf cannot bind to VEGFR1 and NP-1) with VEGF (0.023 nM) and variable VEGFf for 3 h using base parameter values except for the internalization rate for VEGF-bound receptors. Total VEGFR2 bound to VEGF (surface and internal) is shown. B. Similar simulations were performed using all base parameter values but the initial conditions were altered to have 12,000 VEGFR1, VEGFR2, and NP-1 (VEGFR1 = VEGFR2 = NP-1), or 12,000 VEGFR1 and NP-1 but 120,000 VEGFR2 (10*VEGFR2), or 12,000 VEGFR2 and NP-1 but 120,000 VEGFR1 (10*VEGFR1), or 12,000 VEGFR1 and VEGFR2 but 120,000 NP-1 (10*NP-1). Model 1 is open circles and Model 2 is the filled circles in both A and B.

Our model assumed that endocytosis was equivalent for receptors regardless of receptor occupancy. However, many ligand-receptor systems show enhanced endocytosis when ligand is bound. Thus, we tested how varying the relative endocytosis rates would impact VEGF-VEGFR2 retention. As shown in Figure 8, increasing the rate of endocytosis for VEGF-bound receptors resulted in a significant increase in VEGF-VEGFR2 retention while reducing that rate lead to an overall reduction in VEGF-VEGFR2 retention. How these differences might influence signaling however is unclear. Retention includes all VEGF-VEGFR2 complexes (internal and cell surface). If one looked only at surface interactions at 3 hr, the opposite results would be found (data not shown). At 100 times the baseline internalization rate (100*kint), there are essentially no complexes on the surface while at the reduced internalization rate (0.01*kint) there was essentially no internalized complexes with all VEGF-VEGFR2 being on the cell surface.

We further explored the role of receptor levels by varying the levels of VEGFR1, VEGFR2, and NP-1. For example, recent work has suggested that receptor levels may be much lower than NP-1 levels in certain cells [26]. Setting all levels to 12,000 sites per cell, we found that VEGFf resulted in increased VEGF-VEGFR2 retention by cells with Model 2 but only a small decrease with Model 1 (Figure 8B). Increasing the levels of VEGFR1 or VEGFR2 resulted in a change in the retention levels but the overall trend of increasing binding with VEGFf for Model 2 only was found. Increasing NP-1 levels essentially eliminated the effect of VEGFf on VEGFR2 retention, further illustrating the importance of this component for VEGFf activity.

### Conversion of VEGF to VEGFf affects Cell Migration

**Figure 9:**
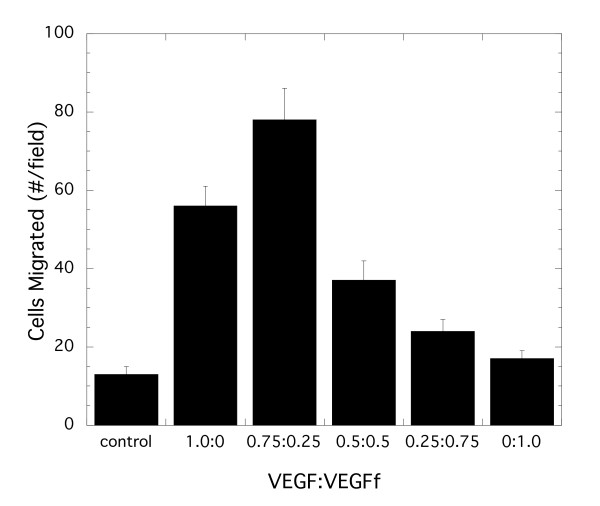
**BAE cell migration towards VEGF and VEGFf**. BAECs were seeded on the upper membrane of transwell cell migration chambers. VEGF and VEGFf were added to the lower chamber in various ratios, with the total amount of VEGF/VEGFf combination or alone being 0.45 nM. The cells were incubated for 2 h at 37°C. Migrated cells were fixed, stained with propidium iodide and images collected by a fluorescent microscope (100 × magnification). Each representative image showed one sixth of the field and were used for quantification. The average cell number migrated ± SEM of 12 fields per condition; n = 3. ANOVA followed by the multi-comparison t-tests revealed significant differences between all groups except the no VEGF "Control" and the all VEGFf "0:1.0" conditions.

NE action on the VEGF system would be predicted to cause a reduction in VEGF binding sites in the ECM and the conversion of VEGF to VEGFf. Simulations indicated that the loss of ECM sites would likely have a negligible effect under standard cell culture conditions and so we focused on evaluating the effect of VEGF conversion to VEGFf. Stimulation of endothelial cell migration is a hallmark of VEGF treatment and is thought to reflect a key component of VEGF's biological function [1]. Thus, we conducted experiments to evaluate whether VEGFf impacted this process. Endothelial cells were plated on microporous transwell inserts and VEGF, VEGFf, or combinations of the two were introduced into the lower chamber. Endothelial cells did not migrate in response to VEGFf alone (*p *= NS) while showing a strong migratory response after 2 h to VEGF (Figure 9). Combinations of VEGF and VEGFf showed a biphasic response with 25% VEGFf and 75% VEGF leading to a statistically significant increased response compared to 100% VEGF while higher ratios of VEGFf led to a diminished response, a not necessarily straightforward result.

### A Simple Increase in VEGF-VEGFR2 is Not Likely Responsible for VEGFf-mediated Enhanced Cell Migration

**Figure 10:**
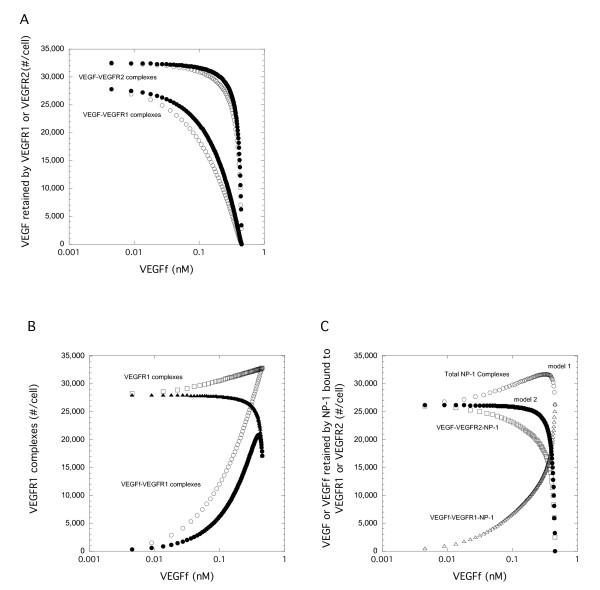
**VEGF Conversion to VEGFf Impacts Binding to VEGFR1 and VEGFR2**. Simulations were run for 2 h using baseline parameter values. The total concentration of VEGF and VEGFf was kept constant at 0.45 nM and VEGFR1, VEGFR2, and NP-1 equal at 12,000 #/cell. (A) VEGF bound to VEGFR2 or VEGFR1 for Model 1 (VEGFf can bind to VEGFR1 alone or when VEGFR1 is bound to NP-1 -open) and Model 2 (VEGFf cannot bind to VEGFR1 bound to NP-1 - filled) are shown. (B) VEGFR1 binding by VEGFf (Model 1- open circle, Model 2 -filled circle), or the sum of both VEGF and VEGFf bound to VEGFR1 (Model 1 - open square, Model 2 -filled triangle) is plotted. (C). NP-1 complexes increase for Model 1. VEGF-VEGFR2-NP-1 complexes (Model 1-open square), VEGF-VEGFR1-NP-1 (Model 1 - open triangle), and the sum of VEGF-VEGFR1-NP-1 and VEGF-VEGFR2-NP-1 complexes (Model 1 -open circle, Model 2 - filled circle). Note that there are no VEGF-VEGFR1-NP-1 complexes with Model 2.

To evaluate various possible mechanisms for the enhanced cell migration observed when portions of VEGF were substituted with VEGFf, simulations were performed for 2 h where the sum of VEGF and VEGFf added was kept constant (0.45 nM) such that at high VEGF concentrations there are low VEGFf levels and at low VEGF concentrations there are high VEGFf concentrations. These conditions might simulate a situation where there is both VEGF and VEGFf entering a cell environment due to local conversion of VEGF to VEGFf by NE rather than the case where excess VEGFf enters a cell environment. VEGFR1, VEGFR2, and NP-1 were all set to the same value (12,000 #/cell). As shown in Figure 10A, with both Models the conversion of VEGF to VEGFf resulted in a reduction in VEGF-VEGFR2 complexes. Even with Model 2, where VEGFf binding to VEGFR1 releases NP-1 for VEGF-VEGFR2 complex stabilization, the reduction in available VEGF for binding to VEGFR2 as a result of conversion to VEGFf is not compensated for by NP-1-mediated stabilization. Similarly, VEGF-VEGFR1 complexes are also reduced by the conversion of VEGF to VEGFf in both cases.

### Signaling by VEGFf is one Possible Mechanism

There is evidence that VEGF signaling can occur through both VEGFR1 and VEGFR2 and that some type of interplay or balance between the two may be at work. For example, we recently showed that VEGF stimulates phosphorylation of both ERK1/2 and Akt in endothelial cells while VEGFf addition resulted in strong phosphorylation of Akt but not ERK1/2[14]. Looking at VEGFR1 binding, we find with Model 1 an increase in complex formation (VEGF + VEGFf) while Model 2 shows a decrease in total VEGFR1 complexes (Figure 10B). This difference reflects the binding of VEGFf to VEGFR1-NP-1 complexes permissible with Model 1. NP-1 has been shown to have a role in VEGF signaling and to alter VEGFR2 signaling [27-29]. We therefore looked at how the combined signaling potential of VEGF and VEGFf through NP-1 in our simulations might proceed as one possible mechanism (Figure 10C). With Model 1, VEGF conversion to VEGFf led to a very strong increase in the total amount of VEGFf-VEGFR1-NP-1 complexes. This counterbalanced the decrease in VEGF-VEGFR2-NP-1 complexes resulted in a combined "total" that was biphasic. In contrast, Model 2 does not allow for VEGF-VEGFR1-NP-1 complexes resulting in only a decreasing VEGF-VEGFR2-NP-1 "signal".

## Discussion

VEGF has long been recognized as an endothelial cell survival factor and mitogen as well as having a role in endothelial cell migration and vessel repair. There are multiple VEGF isoforms with VEGF_165_, a heparin-binding variant, being the most widely expressed *in vivo*. We have recently observed that neutrophil elastase can cleave VEGF and generate VEGFf, a biologically-active fragment of VEGF with altered receptor binding and activity [14]. Unlike several of the other proteolytically processed forms of VEGF, which generally involve truncation of the primary VEGF polypeptide chain [27-29], NE appears to cleave the VEGF chain within internal regions in addition to the termini. In this way, VEGFf is held together by intra- and inter-chain disulfide bonds and shows interesting features resulting from selective alterations in its binding properties. The present paper focuses on coupling computer simulations with experimental studies to delve further into the impact of NE on the VEGF network in order to gain insight on the larger question of how proteolytic damage to growth factor-laden tissues during injury and inflammation might modulate bioavailability and activity.

Enzymatic processing of the extracellular matrix, a depository site for important biological molecules, can result in the release of stored growth factors thereby increasing their availability for activity. In addition, the degradation of the ECM may alter its ability to capture additional growth factors. We show this to be the case with NE and VEGF. Treatment of both surface-deposited fibronectin and cell-derived ECM with NE resulted in a significant decrease in VEGF binding (Figure 2). We postulated that elastase damage to ECM might result in increased cellular VEGFR binding due to a reduction in competition from the ECM sites. Using simulations, however, we found that the impact was negligible unless the sites were of very high number or high affinity (comparable or better than the receptors for VEGF, data not shown) (Figure 3). While the effect of ECM competition was negligible under the *in vitro *cell culture conditions used in our study, it is likely that this process would be very relevant in matrix-rich tissues *in vivo *where VEGF is stored and binding sites are abundant such as in the lung and blood vessel wall. Under those conditions, our simulations suggest that degradation of sites would have a major impact on VEGF availability for receptor binding.

Our studies suggest that a more important consequence of NE treatment with regard to the VEGF system may be the conversion of VEGF to VEGFf. Simulations demonstrate that the presence of VEGFf, with its altered binding properties compared to VEGF, could have a significant impact on VEGF receptor binding. Previously we have shown that VEGFf does not bind to VEGFR2 [14] and data included herein show a loss of binding to NP-1 as well as to fibronectin (Figure 4). We postulated that VEGFf addition would result in increased VEGF binding to VEGFR2 due to a reduction in VEGF-VEGFR1 complexes as a simple consequence of VEGFf-VEGFR1 binding. Using simulations, however, we find that VEGFf can impact VEGF receptor interactions in a more complex manner (Figure 5). When VEGFf binding to VEGFR1 did not prevent VEGFR1 interactions with NP-1 or VEGFf was able to bind to VEGFR1 bound to NP-1 (Model 1), VEGFf did not increase overall VEGF binding to VEGFR2 and had only a limited effect. In contrast, when VEGFf binding to VEGFR1 prevented VEGFR1 interactions with NP-1 (Model 2), VEGFf essentially acted as a release mechanism for the VEGF-VEGFR2 stabilizer, NP-1, and increased VEGF interactions with VEGFR2 were evident.

The role of VEGFf appears therefore to be more of an indirect regulator of VEGF through NP-1 than a direct regulator of VEGF-receptor interactions. This was illustrated further by looking at the impact of NP-1 density (Figure 6) and the coupling rate between NP-1 and VEGFR1 (Figure 7). This type of ternary regulation is not unique to the VEGF system. Many heparin-binding growth factors, such as fibroblast growth factor -2 (FGF-2), form stabilizing complexes with cellular receptors and HSPGs, resulting in significant enhancement of receptor activation. Certainly receptors such as gp130 have been shown to play an important role in cytokine signaling within a number of systems making it a potentially more important system regulator than any individual component. This ternary complex regulation may also have a role in heterodimerization and cross-talk within (i.e., VEGFR1-R2 heterodimers) and between growth factor systems such as what is suggested for the IGF-I and EGF families [30].

Our original hypothesis was that elastase conversion of VEGF to VEGFf would result in the generation of a non-stimulating form of the growth factor due to its inability to bind the major signaling receptor VEGFR2. Both our experimental and simulation results suggest that this model is an oversimplification of this complex ligand-receptor system. VEGF alone stimulates endothelial cell migration while VEGFf alone does not (Figure 9). Conversion of VEGF to VEGFf should result in increased VEGFf and decreased VEGF with the same overall concentration of total growth factor to be found. With cell migration studies designed to test this effect, we found an increase in migration when the VEGF concentration was reduced by 25% with a corresponding substitution of VEGFf, in contrast to what one might expect based on the results with 100% VEGF and 100% VEGFf. Further increases in VEGFf beyond 25% and corresponding decreases in VEGF resulted in reduced migration. Simulations showed that this biphasic response could not be explained simply by an increase and then decrease in VEGF binding to VEGFR2 caused by VEGFf addition (Figure 10) regardless of whether VEGFf bound to VEGFR1-NP-1 complexes or not.

There is however evidence that NP-1 [7] and VEGFR1 are also involved in VEGF signaling [31] and may be involved in mediating the migration process. Previously we found that Akt but not ERK 1/2 was activated by VEGFf suggesting that this key signal pathway might be mediated through VEGFR1 [14]. Using simulations, we find that under conditions where the total VEGF plus VEGFf remains constant, VEGF binding to VEGFR1 and VEGFR2 is reduced while VEGFf binding to VEGFR1 is increased (Figure 10). Further, when one focuses on NP-1 stabilized VEGF and VEGFf complexes, we find that, with Model 1, a biphasic binding situation exists with a peak sum of VEGF-VEGFR2-NP-1 and VEGF-VEGFR1-NP-1 at an intermediate combination of VEGF and VEGFf. Certainly this is not direct evidence but simply further illustrates how NP-1 might be critical to the regulation of VEGF-VEGFf activity. Further experimental studies are needed to determine exact mechanisms but our simulations suggest possibilities worth exploring with regard to NP-1. There are also recent reports indicating that VEGFR1 and VEGFR2 can form heterodimers [32]. Thus, VEGFf also has the potential to influence the formation of these complexes directly and through NP-1 interactions. As experimental data become available it will be interesting to explore these additional aspects of the system to fully appreciate how its complexity can be used to produce sophisticated modes of regulation.

## Conclusions

We have shown that VEGFf, an elastase-generated product of VEGF, has a biphasic impact on VEGF stimulated migration and, using simulations, we postulate that this effect is via VEGFf-mediated VEGFR1 stimulation. Simulations suggest that VEGFf activity is controlled by its interactions with NP-1 and VEGFR1 and that release or restriction of NP-1 controls whether enhanced VEGF binding of VEGFR2 is likely to occur. Further experimental studies targeted at testing the simulation predictions are necessary to fully understand the complex mechanisms controlling VEGF-signaling. Moreover, these studies suggest that simulations can be a valuable means to interpreting the complexity of perturbations within a complex multi-receptor system and highlight the need for quantitative parameter measurements to obtain mechanistic understanding.

## Authors' contributions

KFW developed and performed the computational simulations. EK performed the experimental studies. MAN was involved in experimental and simulation design as well as data analysis. All authors read and approved the final manuscript.
